# Use of troponin assay after electrical injuries: a 15-year multicentre retrospective cohort in emergency departments

**DOI:** 10.1186/s13049-021-00955-6

**Published:** 2021-09-26

**Authors:** Douillet Delphine, Kalwant Stéphanie, Amro Yara, Gicquel Benjamin, Arnaudet Idriss, Savary Dominique, Le Bastard Quentin, Javaudin François

**Affiliations:** 1 Département de Médecine d’Urgence, Centre Hospitalier Universitaire d’Angers, 4 rue Larrey, 49100 Angers, France; 2 UMR MitoVasc CNRS 6015 - INSERM 1083, Health Faculty, Univ of Angers, FCRIN, INNOVTE, Angers, France; 3 Emergency Department, Nantes University Hospital, Nantes, France; 4 Microbiotas Hosts Antibiotics and Bacterial Resistances (MiHAR), University of Nantes, Nantes, France; 5 EHESP, Irset, Inserm, UMR S1085, CAPTV CDC, University of Rennes, Rennes, France

**Keywords:** Electric injuries, Troponin, MACE, Emergency Department, Cardiac arrhythmias

## Abstract

**Background:**

Patients with electrical injury are considered to be at risk of cardiac arrhythmia. Assessing the risk of developing a major adverse cardiac event (MACE) is the cornerstone of patient management. The aim of this study was to assess the performance of initial troponin and troponin rise to predict Major Adverse Cardiac Events (MACEs) in all patients with electrical injuries admitted to the Emergency Department.

**Methods:**

This is a multicentre retrospective study in which consecutive patients with electrical injuries admitted to the Emergency Departments (ED) (adult and paediatric) of five French Hospitals were included between 2005 and 2019. The threshold for troponin elevation is based on the European Society of Cardiology guidelines for patients presenting without persistent ST segment elevation. The primary endpoint was the rate of MACE.

**Results:**

A total of 785 included patients were admitted to ED with a first diagnosis of electrical injury during the study period. Troponin assays were performed in 533 patients (67.9%), including 465 of 663 adults (70.1%) and 68 of 122 children (55.7%) and 17/533 (3.2%) of patients had an initial elevated troponin. If none of the clinical criteria for MACE were present (i.e., previous known heart disease, exposure to a high voltage of ≥ 1000 Volts, initial loss of consciousness, or an abnormal initial ECG), this defined a low-risk subgroup (n = 573, 76.0%) that could be safely discharged. The initial positive troponin assay had a sensitivity of 83.3 (95% CI 35.9–99.6%), a specificity of 97.7 (95% CI 96.1–98.8%), a positive likelihood ratio 36.6 (95% CI 18.8–71.1%) and a negative predictive value of 99.9 (95% CI 99.2–99.9%) in predicting a MACE.

**Conclusions:**

Troponin assay appears to be a predictive marker of MACE risk and should be considered in high-risk patients.

**Supplementary Information:**

The online version contains supplementary material available at 10.1186/s13049-021-00955-6.

## Background

Electrical injuries can cause immediate respiratory and cardiac arrest as well as heart failure, cardiomyopathy and myocardial infarction [1–3]. However, electrical injuries are very varied and heterogeneous, ranging from minor injuries or burns to life-threatening damage to internal organs or significant long-term morbidity [1, 4–7]. The two major cardiac complications of electrical shock are arrhythmias and myocardial tissue injuries. These cardiac events are infrequent following an electrical injury, especially in cases of low voltage (mortality rates = 0–2.6%) [8, 9]. Most cardiac events occur immediately after the accident resulting from the proarrhythmic effect of electric shock, but delayed ventricular arrhythmias have also been reported in rare cases [10]. Most studies did not report a delayed events [11–14].

The major issue in electrical accidents lies primarily in the risk stratification of developing a serious cardiac event in order to identify a large subgroup of low-risk patients where rapid discharge is safe and of those at a high-risk requiring Electrocardiogram (ECG) monitoring in an intensive care unit. ECG on admission appears to be the most predictive element of cardiac complications. Most cardiac arrhythmias in patients following electrical injury can be diagnosed by an initial ECG [1, 15]. The risk of serious cardiac arrhythmia is considered high in patients corresponding to four main criteria: previous known heart disease and/or exposure to a high voltage ≥ 1000 Volts or/and with initial loss of consciousness and/or an abnormal initial ECG [1, 15–17]. Troponin assays are controversial following electrical injury. In the practical algorithm of the recommendations published in *Eur Heart Journal* in 2018, it is suggested to perform a systematic assay of troponin and monitoring of troponin elevation. The text highlightsthat "no significant studies exist concerning the usefulness of troponin in these clinical situations" [1]. However, other authors do not recommend it in routine practice for all patients due to the lack of assessment [18, 19]. The European Resuscitation Council (ERC) guidelines do not mention the usefulness of biological markers after an electrical injury in special circumstances [20].

### Study aims

The main aim of this study was to assess the performance of initial troponin and troponin elevation in order to predict Major Adverse Cardiac Events (MACEs) in all patients with electrical injuries admitted to the ED. Our population of interest did not include the most serious patients with major trauma, severe burns or direct admission to the ICU. The secondary objective was to evaluate the appropriateness of troponin assays in subgroups classified as low risk and those classified as high risk by clinical and ECG evidence. We aimed to isolate a group of low-risk patients who could be rapidly and safely discharged from the ED by clinical criteria without the need for biological testing. We hypothesised that troponin could identify patients at risk of MACE after an electrical injury.

## Methods

### Study design and setting

This is a multicentre retrospective study. Consecutive patients with electrical injuries admitted to the Emergency Departments (ED) (adult and paediatric) of five French Hospitals were included. From these five centres, two EDs are University Hospital Centres (average ED admissions in 2019: 100,000 (70,000 in the adult department and 30,000 in the paediatric department), one is a High-Capacity Hospital Centre (average admissions in 2019 > 60,000) and two are Low-Capacity Hospital Centres (average admissions in 2019 < 60,000). These 5 hospitals cover a defined population in the west of France. In these ED, patients older than 16 years are referred to the adult ED managed by emergency physicians, and younger patients are referred to the paediatric department managed by paediatricians. None of the 5 centres had a well-established protocol for the management of patients suffering from electrical injuries. The STARD recommendations were followed for the reporting of diagnostic studies [21].

Firstly, we will describe the outcome of the entire cohort. Secondly, we will assess the predictive value of troponin in patients who had a troponin assay. Thirdly, we will perform a subgroup analysis according to risk factors to position troponin testing in a relevant as possible wat in the management of patients.

### Participants

We extracted all discharges records of ED patients from a 15-year period (2005–2019) with the corresponding ICD-10 codes as the principal diagnosis (“Appendix”). The most affected victims (i.e., out of hospital cardiac arrest, extensive burns, severe rhythmic disorders, comatose patients or early MI) were treated outside the hospital by an Emergency Medical Service (EMS) and were not included in this study because they were referred directly to an intensive care unit (ICU) or an operating room without being admitted to the ED. Clinical data were obtained from the hospital information system and patient records. Baseline demographics, medical history, and antiarrhythmic medication were registered along with the location, time, and circumstances of the electrical injury. Furthermore, all clinical parameters which are deemed to be risk factors for cardiac arrhythmias based on the ERC criteria were summarised. We also recorded presenting symptoms, severity of burns, and other injuries.

### Measurements

The threshold for troponin elevation is based on the European Society of Cardiology guidelines for patients presenting without persistent ST segment elevation as there are no specific prospective data available on this specific population affected by electrical accidents [22]. From 2005 to 2011, the laboratories of the five centres measured troponin T and the threshold was set at 0.03 μg l^−1^ according to the manufacturer’s guidelines; from 2011, high-sensitivity troponin T was used. We used the manufacturer’s recommended 99th percentile upper reference limit (URL) to reduce site-to-site variability when determining the cut-off point. A significant increase in troponin was defined as an increase of at least 10 ng/l within 6 h or 6 ng/l within 3 h as per the manufacturer’s guidelines.

### Outcomes

The primary endpoint was the rate of Major Adverse Cardiac Events (MACEs) from admission to the ED until discharge or during hospitalisation or upon re-presentation to the ED or in another department of the referral hospital within 30 days. MACE was a composite measure defined as (i) acute myocardial infarction according to the Fourth Universal Definition of Myocardial Infarction, Myocardial Infarction with No Obstructive Coronary Arteries (MINOCA), myocarditis or Tako-Tsubo Syndrome [23], (ii) sustained Ventricular Tachycardia (VT) or Ventricular Fibrillation (VF) according to the definition provided by the American Heart Association [24], (iii) in-hospital cardiac arrest with return of spontaneous circulation (ROSC), (iv) death from any cause. Patients diagnosed with unstable angina or myocardial injuries not meeting the previously stated criteria were not considered as having suffered a MACE.

The secondary endpoint was the rate of cardiac events during in-hospital monitoring or upon re-presentation to the ED or in another department of the referral hospital within 30 days. A cardiac event includes MACE and the need for cardiac treatments (revascularisation and/or cardiac medication), non-sustained VT, arrythmias including sinus tachycardia (more than 30 min), atrial tachyarrhythmia or bradyarrhythmia requiring monitoring and/or treatment.

#### Ethics

Due to the non-interventional retrospective nature of the current study no informed consent was required (Deliberation no. 2016-262, 2016-263, CNIL MR-003). Ethical approval for this study was obtained from the Nantes Research Ethics Committee (Groupe Nantais d’Ethique dans le Domaine de la Santé, GNEDS).

#### Statistical analysis

Continuous variables are presented with their median, first and third quartiles (Q1–Q3). Categorical variables are summarised with the number of patients and percentage with a 95% confidence interval (95% CI). The Chi-squared test, Fisher’s exact test, Student’s t-test and the Mann–Whitney U test were used when appropriate (two-tailed; level of significance p < 0.05).

To assess the performances of the initial troponin aim 7 say and the second troponin assay for predicting the primary endpoint, we assessed diagnostic performances (sensitivity, specificity, negative and positive predictive values, negative and positive likelihood ratio and accuracy).

Performances of the 4 high-risk clinical items combined (with prior known heart disease and/or exposure to a high voltage of ≥ 1000 Volts and/or with initial loss of consciousness or/and an abnormal initial ECG) were assessed to predict MACE. This item is considered as positive if one or more item was present. The aim is to identify simple clinical criteria to isolate a group of patients who will not have MACE (i.e., < 1%) without the need for a bioassay, for a safe ED rule out.

A subgroup analysis was performed in low-risk patients and in high-risk patients using the same endpoint. Patients without ECG at baseline were excluded from this analysis.

All statistical analyses were 2-tailed, and a *p* value less than 0.05 was required for statistical significance. No imputation of missing data was performed. Since occurrences of electrical injuries are rare, we did not determine a necessary sample size. All statistical analysis was performed using R Statistical Software (version 4.0.3; R Foundation for Statistical Computing, Vienna, Austria) (URL https://www.R-project.org/).

## Results

**Fig. 1:**
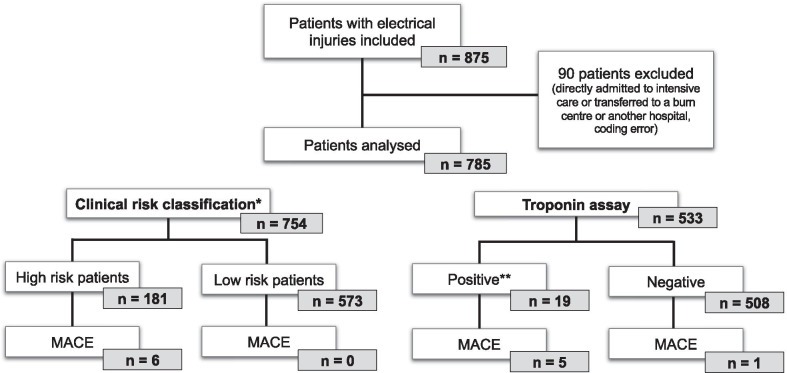
Flow diagram. *According to the 4 high-risk clinical items combined (previous known heart disease, exposure to a high voltage of ≥ 1000 V, initial loss of consciousness, abnormal initial ECG); if ≥ 1 items = high risk patients. Missing data, n = 31. **Initial elevated troponin and/or significant rise on the 2nd essay. *MACE* Major Adverse Cardiac Even

A total of 875 patients were admitted to hospitals with a first diagnosis of electrical injuries during the study period. Ninety were excluded for various reasons which are summarised in Fig. 1. The population incidence for patients severely injured admitted directly to ICU was 0.21 per 100,000 person-years (95% CI 0.17–0.26) and of 1.87 per 100,000 person-years (95% CI 1.74–2.00) for moderate or less injured patients admitted to the ED. The final analysis assessed 785 patients (663 adults (84.5%) and 122 children (15.5%)) (Table 1). The sex ratio was 0.33 (193 women/59 men2) and the median age was 30 years.

**Table 1:** Demographic data and management of patients with electrical injuries

	All patients (n = 785)	Adults (n = 663)	Children (n = 122)
*Demographic characteristics*			
Male sex—no. (%)	592/785 (75.4)	511/663 (77.1)	81/122 (66.4)
Age—year, median (IQR)^a^	30 (21–39)	31 (25–42)	5 (2–9)
*Medical history*—*no. (%)*			
Cardiovascular risks factors (≧ 1)	68/629 (10.8)	68/545 (12.5)	0/84
Diabetes	10/629 (1.6)	10/545 (1.8)	–
Dyslipidaemia	20/629 (3.2)	20/545 (3.7)	–
Smoking	34/629 (5.4)	34/545 (6.2)	–
Hypertension	15/629 (2.4)	15/545 (2.8)	–
Overweight	7/629 (1.1)	7/545 (1.3)	–
Family history	1/629 (0.2)	1/545 (0.2)	–
Cardiac history	25/768 (3.3)	23/649 (3.6)	2/119 (1.7)
Coronaropathy	13/768 (1.7)	13/649 (2.0)	0
Heart arrhythmia	9/768 (1.2)	8/649 (1.2)	1/119 (0.8)
Valvulopathy	3/768 (0.4)	3/649 (0.5)	0
Other (interventricular communication, …)	6/768 (0.8)	5/649 (0.8)	1/119 (0.8)
*Type of electrical Injury*—*no. (%)*			
Trauma context			
Workplace	254/559 (45.4)	254/464 (54.7)	0
Domestic accident	301/559 (53.8)	232/464 (50.0)	69/95 (100)
Suicide attempt	4/559 (0.7)	4/464 (0.9)	0
High voltage (> 1000Volts)—no. (%)	55/568 (9.7)	53/493 (10.8)	2/75 (2.7)
Transthoracic current—no. (%)	273/459 (59.5)	238/395 (60.3)	35/64 (54.7)
*Symptoms*—*no. (%)*			
Asymptomatic^a^	234/785 (29.8)	203/663 (30.6)	31/122 (25.4)
Initial loss of consciousness	38/754 (5.0)	35/636 (5.5)	3/118 (2.5)
Burns	99/756 (13.1)	76/636 (11.9)	23/120 (19.2)
Second and third degree	52/756 (6.9)	35/636 (5.5)	17/120 (14.2)
TBSA ≦ 1% (second and third degree)	44/728 (6.0)	28/619 (4.5)	16/109 (14.2)
Chest pain	80/744 (10.8)	77/625 (12.3)	3/119 (14.7)
Headache and/or vertigo	40/744 (5.4)	37/625 (5.9)	3/119 (14.7)
Numbness of extremities	69/744 (9.3)	63/625 (10.1)	6/119 (5.0)
Association with traumatic injury	95/744 (12.8)	88/625 (14.1)	7/119 (5.9)
Initial HR—bpm*^d^	80 ± 20.1	78 ± 17.3	103 ± 22.7
Initial systolic blood pressure—mmHg	130 ± 17.3	131 ± 16.3	110 ± 12.3
Initial diastolic blood pressure—mmHg	79 ± 11.6	80 ± 11	69 ± 11.8
*Management*			
ECG on admission	756/785 (96.3)	641/663 (96.7)	115/122 (94.3)
Abnormal ECG	99/756 (13.1)	91/641 (14.2)	8/115 (7.0)
Troponin assay	533/785 (67.9)	465/663 (70.1)	68/122 (55.7)
Elevated initial troponin	17/533 (3.2)	16/465 (3.4)	1/68 (1.5)
Second troponin assay	197/533 (37.0)	183/465 (39.4)	14/68 (20.6)
Troponin rise	8/533 (1.5)	8/465 (1.7)	0/68 (0)
Delay of the second assay—hs, median (IQR)^b^	6 (4–11)	6 (4–11)	6 (4–12)
Type of monitoring			
Scope < 6 h	41/559 (7.3)	38/489 (7.8)	3/70 (4.3)
Scope > 6 h and < 24 h	125/559 (22.4)	106/489 (21.7)	19/70 (27.1)
Scope > 24 h	27/559 (4.8)	26/489 (5.3)	1/70 (1.4)
ECG each 6 h without scope	14/559 (2.5)	13/489 (2.7)	1/70 (1.4)
ECG > 6 h without scope	33/559 (5.9)	28/489 (5.7)	5/70 (7.1)
Length of monitoring—hours, mean (SD)^c^	15.5 ± 23	13.5 ± 23	24 ± 19
Hospitalisation	420/715 (58.7)	333/614 (54.2)	87/101 (86.1)
Intensive care unit	103/715 (14.4)	95/614 (15.5)	8/101 (7.9)
Short term hospitalisation unit	182/715 (25.5)	176/614 (28.7)	7/101 (6.9)
Conventional medical service	65/715 (9.1)	36/614 (5.9)	28/101 (27.7)
Discharge from the ED	365/715 (51.0)	330/614 (53.7)	35/101 (34.7)

*IQR* interquartile range, *TBSA* total body surface area, *HR* heart rate—beats per minute

^a^No missing data

^b^Missing data (n = 53)

^c^Missing data (n = 226)

^d^Missing data (n = 129)

An ECG was performed in 756 patients (96.3%) and results were abnormal in 99 cases (12.6%) with the occasional association of several anomalies (Table 1). The most frequent electrocardiographic observations were non-specific ST-T changes (n = 47/99, 47.5%), incomplete right bundle branch block (n = 27/99, 27.3%), sinus tachycardia (> 100 bpm) (n = 16/99, 16.2%), first-degree atrioventricular block (n = 6/99, 6.1%), ventricular extrasystole (n = 5/99, 5.1%), significant ST-elevation (n = 4/99, 4.0%), sinus bradycardia (< 60 bpm) (n = 3/99, 3.0%), ventricular pre-excitation syndrome (n = 1), non-sustained VT (n = 1).

Troponin assays were performed in 533 patients (67.9%), of which 465 of 663 were adults (70.1%) and 68 of 122 were children (55.7%). An initial elevated troponin was observed in 17/533 (3.2%) of patients. In total, 197/533 patients (37.0%) received a new troponin assay at a median of 6 [4–11] hours after the first assay. Eight presented an increase of the initial troponin (including 6 patients with initial positive troponin).

Six patients suffered a MACE (6/785, 0.76% (95% CI 0.04–1.66%) (Additional file 1: Table S1). Among them, 3 suffered VF/VT with subsequent fatal cardiorespiratory arrest (one had a STEMI on the initial ECG), 2 had sustained VT and one patient had a Tako-Tsubo diagnosis according to the International Takotsubo Diagnostic Criteria [25]. The overall patient mortality was 0.38% (95% CI 0.13–1.12%) consisting of which 2 adults n = 2/663 (0.30%, 95% CI 0.08–1.09%) and one child n = 1/122 (0.82%, 95% CI 0.14–4.5%). All MACE or deaths occurred during the initial ED work-up (< 24 h) and none during hospitalisation. A total of 13 patients suffered a cardiac event, including patients with MACE (Additional file 2: Table S2).

In the univariate model, age, cardiac history, initial loss of consciousness, transthoracic current, abnormal initial ECG, positive initial troponin assay and troponin elevation were significantly associated with MACE occurrence (Table 2).

**Table 2:** Patient characteristics by MACE occurrence

	No MACE n = 779 (%)	MACE n = 6 (%)	*p* value
Age (years)—median (IQR)	42 ± 11.2	53 ± 31.9	< 0.001
Cardiovascular risks factors (≧ 1)—no. (%)	65 (8.3)	3 (50.0)	0.01
Cardiac history—no. (%)	22 (2.8)	3 (50.0)	< 0.001
High voltage (> 1000 Volts)—no. (%)	54 (6.9)	1 (16.7)	0.42
Initial loss of consciousness—no. (%)	35 (4.5)	3 (50.0)	< 0.001
Transthoracic current—no. (%)	267 (34.3)	6 (100.0)	< 0.001
Symptomatic—no. (%)	545 (70.0)	6 (100.0)	0.04
Chest Pain—no. (%)	107 (13.7)	1 (16.7)	0.84
Headache or vertigo—no. (%)	62 (8.0)	1 (16.7)	0.49
Burns—no. (%)	128 (16.4)	3 (50.0)	0.27
Abnormal ECG—no. (%)	94 (12.1)	5 (83.3)	< 0.001
Positive troponin—no. (%)	12 (1.5)	5 (83.3)	< 0.001
Troponin rise—no. (%)	6 (0.8)	2 (33.3)	< 0.001

*MACE* major adverse cardiac event, *IQR* interquartile range, *OR* odds ratio, *95% CI* 95% confidence interval, *ECG* electrocardiogram

### Troponin performances

Prognostic performances of the initial positive troponin assay to predict MACE are summarised in Table 3. Among the patients with initial positive troponin (n = 17), 5 suffered a MACE (29.4%) and among them two had abnormal ECG on admission (significant ST-elevation). To predict a MACE, the initial positive troponin assay had a sensitivity of 83.3 (95% CI 35.9–99.6%), a specificity of 97.7 (95% CI 96.1–98.8), a positive likelihood ratio of 36.6 (95% CI 18.8–71.1%) and a negative predictive value of 99.9 (95% CI 99.2–99.9%) (Table 4). The troponin rise had a sensitivity of 33.3 (95% CI 4.3–77.7%), a specificity of 99.2 (95% CI 98.3–99.7%), a positive likelihood ratio of 43.3 (95% CI 10.8–172.7%) and a negative predictive value of 99.5 (95% CI 99.1–99.7%). Of the patients with an initially elevated troponin, only one was asymptomatic. The patient did not receive troponin monitoring and was monitored for 24 h without the occurrence of a MACE.

**Table 3:** Diagnostic performances for the prediction of a major adverse cardiac event

For prediction of MACE in the whole population (n = 533 patients*)	The 4 high-risk clinical items	Initial positive troponin assay	Troponin rise
Sensitivity, % (95% CI)	100.0 (54.1–100)	83.3 (35.9–99.6)	33.3 (4.3–77.7)
Specificity, % (95% CI)	76.6 (73.4–79.6)	97.7 (96.1–98.8)	99.2 (98.3–99.7)
*Predictive value, % (95% CI)*			
Positive	4.3 (3.8–4.9)	29.3 (17.5–44.6)	24.9 (7.7–56.9)
Negative	100.0 (99.9–100.0)	99.9 (99.2–99.9)	99.5 (99.1–99.7)
*Likelihood ratio (95% CI)*			
Positive	4.6 (4.1–5.2)	36.6 (18.8–71.1)	43.3 (10.8–172.7)
Negative	0	0.17 (0.03–1.02)	0.7 (0.4–1.2)
Accuracy, % (95% CI)	76.9 (73.7–79.8)	97.6 (95.9–98.7)	98.7 (97.7–99.4)

For prediction of MACE in high-risk population (= 153 patients*)	Initial positive troponin assay	Troponin rise
Sensitivity, % (95% CI)	83.3 (35.9–99.6)	33.3 (4.3–77.7)
Specificity, % (95% CI)	95.2 (90.4–98.1)	97.2 (93.5–99.1)
*Predictive value, % (95% CI)*		
Positive	41.7 (24.2–61.5)	28.5 (8.8–62.4)
Negative	99.3 (95.9–99.9)	97.7 (96.1–98.7)
*Likelihood ratio (95%CI)*		
Positive	17.5 (7.8–39.2)	11.8 (2.8–49.0)
Negative	0.2 (0.0–1.1)	0.7 (0.4–1.2)
Accuracy, % (95% CI)	94.8 (90.0–97.7)	95.1 (90.9–97.7)

*Performed in patients with troponin assay

*MACE* major adverse cardiac event, *95% CI* 95% confidence interval

**Table 4:** Contingency table of events according to initial troponin and according to stratification by clinical items

Entire cohort (n = 533)	Occurrence of a MACE	No MACE	Total
Initial elevated troponin (n)	5	12	17
Row percentage (%)	29.41	70.59	100.00
Column percentage (%)	83.33	2.28	3.19
Normal initial troponin (n)	1	515	516
Row percentage (%)	0.19	99.81	100.00
Column percentage (%)	16.67	97.72	96.81
Total	6	527	533
Row percentage (%)	1.13	98.87	100.00
Column percentage (%)	100.00	100.00	100.00

High risk according to 41 (n = 153)			
Initial elevated troponin (n)	5	7	12
Row percentage (%)	41.67	58.33	100.00
Column percentage (%)	83.33	4.76	7.84
Normal initial troponin (n)	1	140	141
Row percentage (%)	0.71	99.29	100.00
Column percentage (%)	16.67	95.24	92.16
Total	6	147	153
Row percentage (%)	3.92	96.08	100.00
Column percentage (%)	100.00	100.00	100.00

### Subgroup analysis

The 4 high-risk clinical items combined separated the low-risk patients (n = 573, 76.0%) from the high-risk patients (n = 181, 24.0%) (Table 4 and Additional file 1: Table S1).

In the low-risk group, no patient had a MACE or died and 4 patients had a cardiac event (3 sinus bradycardia and 1 sinus tachycardia). A total of 65 patients without any symptoms were hospitalised for monitoring. In the whole population the 4 high-risk clinical items combined had a sensitivity of 100 (95% CI 54.1–100.0%), a specificity of 76.6 (95% CI 73.4–79.6%) and a negative predictive value of 100 (95% CI 99.9–100%) to predict or exclude a MACE (Table 3).

In the high-risk group, 6 patients had a MACE (prevalence of 3.3% (95% CI 1.5–7.0%), of which 3 died (prevalence of 1.6% [95% CI 0.6–4.7%]) (i.e., one patient died in the ED and two patients during hospitalisation), and 9 had cardiac events. In the high-risk group, the initial positive troponin assay had a sensitivity of 83.3 (95% CI 35.9–99.6%), a specificity of 95.2 (95% CI 90.4–98.1%) a negative predictive value of 99.3 (95% CI 95.9–99.9%) and a positive likelihood ratio of 17.5 (95% CI 7.8–39.2%).

## Discussion

The occurrence of MACE is rare following an electrical accident. Troponins could be a relevant marker in electrical accidents for predicting MACE. However, the excellent sensitivity and negative predictive value of the “4-high-risk clinical items combined” represent it the first step in risk stratification.

In this study, the troponin assay was performed in 68% of all patients in the ED and in 55.7% of children. Although there are no clear recommendations for adults, some authors do not recommend the use of troponin assays for children, except for victims of high-voltage electrical exposure, lightning strikes, and severe burns [26]. In the absence of clear guidelines, actual practices differ. Indeed, Searle et al. found that troponin assays were being used in 94% in adults and 55% in children, while Bailey et al. found a rate of 64% in high-risk patients [27, 28]. We observed that the patient’s initial risk assessment had little impact on troponin assay with a rate of 66.3% in the low-risk subgroup.

Here, we found elevated troponin in only 2.2% of patients admitted to the emergency department. In a retrospective study, Choi et al., found elevated troponin in 72.9% of patients with cardiac complications (n = 78/107) [29]. Only one child had an elevated troponin level. Searle et al. also reported that the troponin was rarely elevated, with only 2 out of 144 adults and no children exhibiting troponin elevation [27]. Even in high-risk patients, Bailey et al. found that all were at or below the detection limit [28]. However, Gokdemir et al. found 3 children out of 36 with elevated troponin after a low-voltage electrical injury, but this did not affect mortality [30].

In our study, the 4-high-risk clinical items combined which are prior known heart disease and/or exposure to a high voltage ≥ 1000 Volts and/or with initial loss of consciousness and/or an abnormal initial ECG obtained excellent values to exclude the occurrence of MACE (sensitivity 100% and negative predictive value 100%). Only 4 cardiac events occurred but had no negative impact on patients. Using this first step in risk stratification enables the identification of a large low-risk subgroup (76%, n = 573/754) that can be rapidly discharged from the emergency department with only an initial examination and an ECG. Indeed, this strategy would allow a rapid and safe discharge of most patients, which would limit the waiting time of these patients and others by repercussion, and avoid the overcrowding encountered in the ED [31]. These 4 items are those found in the literature and were recently proposed by Waldman [1].

**Fig. 2:**
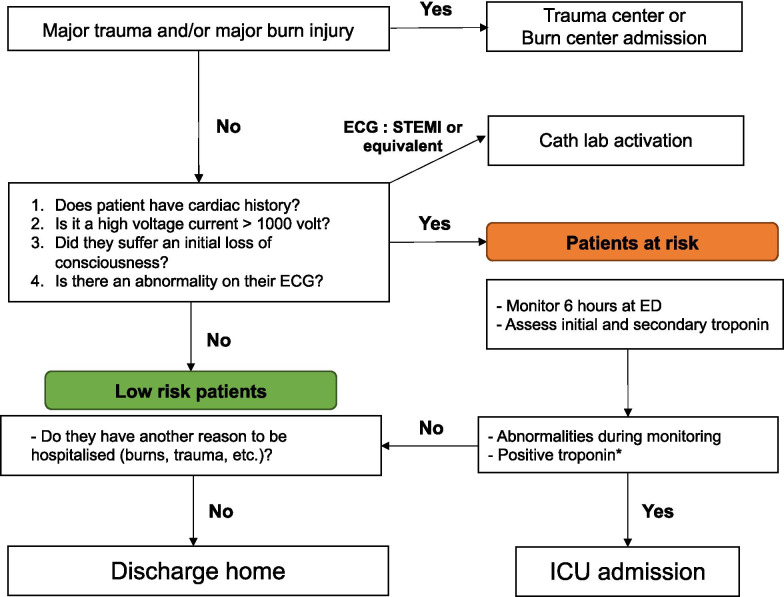
Flowchart for a standard protocol for patients with electrical injury (based on the paper by Waldmann et al. [1]). *No prospective data available, a procedure in line with the acute coronary syndrome is recommended: follow-up 12-channel ECG, troponin after 6 h and, if necessary, after 12–24 h (ERC)

In the whole population, the sensitivity of troponin value in predicting cardiac events was intermediate (83%) but had a large confidence interval due to the low rate of MACE. The same performances were obtained in the high-risk subgroup. Given the high level of specificity, the troponin could identify MACE in all patients that suffer electrical accidents. However, it seems unnecessary to add bioassays in the subgroup of low-risk patients for the performance of the clinical items alone. It may therefore be appropriate to reserve troponin dosing and control for high-risk patients. In this study, we focused only on troponin and not on creatine kinase-MB. Cardiac troponin T has been recognised as the most sensitive and specific cardiac enzyme for the diagnosis of myocardial injury in general. Data indicate that creatine kinase-MB is an unreliable marker for electrical injuries because of an inadequate sensitivity and potential confusion with peripheral skeletal muscle injury [4]. On the basis of this study, a standardised approach is conceivable, combining the ERC recommendations with the criteria evaluated by Blackwell et al. and Waldmann et al. (Fig. 2) [1, 14]. This study does not allow this algorithm to be reliably validated, but it is clear that performing troponins in all patients presenting to the ED without having previously assessed clinical severity items for electrical injuries does not seem useful. Multiplying tests in the ED was associated with prolonged length of stay and an increase in the use of resources [32]. Various strategies for securely decreasing the number of complementary exams are being implemented to combat frequent ED overcrowding [33]. However, the risk assessment in our study was probably biased due to its retrospective design. Indeed, there was no standard of care and it is possible that patients considered at low-risk did not have troponins or ECG. For high-risk patients, a small subgroup of patients could be admitted directly to the ICU.

MACEs occurred during the initial ED workup. This is consistent with previous studies that found a non-existent or low risk of developing MACE at a distance from the electrification injury [10, 12, 13]. In 1986, Purdue et al. asked whether monitoring for all patients was a “necessity or a luxury” and proposed the use of clinical history and ECG as a basis for patient selection [34]. In our study, 65 patients at low risk were hospitalised without any symptoms in order to be monitored.

In our series, we found a case of Tako-Tsubo syndrome after a low voltage electrical injury in a 65-year-old woman. This is a rare case but has already been described in the literature [35]. It is difficult to assess whether the electrical injury was an emotional trigger or a physical trigger [25].

In the context of a rare and heterogeneously managed disease leading to inconsistent guidelines, one of the strengths of this study was its multicentric design and its significant number of patients.

### Limitations

First of all, there were very few cases of troponin elevation and few MACEs occurred, thus limiting the statistical power of the study. However, creating larger databases on this condition appears to be very difficult due to its low prevalence. Secondly, due to the retrospective design of the study many data could not be collected and there may be an information bias. A large-scale prospective study would be required to validate the findings of this retrospective study. The third limitation was the lack of availability of the length of stay in the ED and the delay between the accident and the occurrence of MACE, which resulted in a lack of support for our conclusions regarding the monitoring period. Lastly, it is possible that early discharge patients may have had a secondary MACE but this would potentially have been accounted for in referral hospital records.

## Conclusions

Patients with an electrical injury have a highly variable risk of major adverse cardiac events. It is necessary to perform risk stratification based initially on clinical items and an ECG to safely discharge a large subgroup of patients from the ED. This will also likely help reserve troponin assays, control and patient monitoring for those at high risk.

### Supplementary Information

**Additional file 1: Table S1.** Characteristics of patients who suffered from MACE.

**Additional file 2: Table S2.** Management and outcomes of patients according to their risk classification*.

## Data Availability

Available upon reasonable request.

## Appendix

See Table 5.

**Table 5:** International classification of diseases, tenth edition (Icd-10) diagnostic codes to define electrical injuries

Type	Code	Label
ICD-10	W85	Exposure to electric transmission lines
ICD-10	W86	Exposure to other specified electric current
ICD-10	W87	Exposure to unspecified electric current
ICD-10	W29	Contact with other powered hand tools and household machinery
ICD-10	T75.4	Effects of electric current
ICD-10	T75.0	Effects of lightning

## Abbreviations

95% CI: 95% Confidence Interval
ED: Emergency Department
ECG: Electrocardiogram
EMS: Emergency Medical Service
ERC: European Respiratory Council
ESC: European Society of Cardiology
ICU: Intensive Care Unit
MACE: Major Cardiac Event
MINOCA: Myocardial Infarction with No Obstructive Coronary Arteries
ROSC: Return Of Spontaneous Circulation
STEMI: ST-Elevation Myocardial Infarction
URL: Upper Reference Limit
VF: Ventricular Fibrillation
VT: Ventricular Tachycardia
