# Correction to: Use of troponin assay after electrical injuries: a 15-year multicentre retrospective cohort in emergency departments

**DOI:** 10.1186/s13049-021-00968-1

**Published:** 2021-10-19

**Authors:** Delphine Douillet, Stéphanie Kalwant, Yara Amro, Benjamin Gicquel, Idriss Arnaudet, Dominique Savary, Quentin Le Bastard, François Javaudin

**Affiliations:** 1 Département de Médecine d’Urgence, Centre Hospitalier Universitaire d’Angers, 4 rue Larrey, 49100 Angers, France; 2 UMR MitoVasc CNRS 6015 - INSERM 1083, Health Faculty, Univ of Angers, FCRIN, INNOVTE, Angers, France; 3 Emergency Department, Nantes University Hospital, Nantes, France; 4 Microbiotas Hosts Antibiotics and Bacterial Resistances (MiHAR), University of Nantes, Nantes, France; 5 EHESP, IrsetInserm, UMR S1085, CAPTV CDC, University of Rennes, Rennes, France

## Correction to: Scand J Trauma Resusc Emerg Med (2021) 29:141 10.1186/s13049-021-00955-6

Following the publication of the original article [1] the authors brought to our attention that their first and last names had unfortunately been interchanged.

The correct authors’ names are shown in the author list of this Correction and have already been updated in the original article.
