# Supplementary material for: Use of troponin assay after electrical injuries: a 15-year multicentre retrospective cohort in emergency departments
Source: Scand J Trauma Resusc Emerg Med. 2021 Sep 26;29:141. doi: 10.1186/s13049-021-00955-6 (PMC8474711; doi:10.1186/s13049-021-00955-6)
Supplement: Supplementary file 1 — Additional file 1: Table S1. Characteristics of patients who suffered from MACE. [file 13049_2021_955_MOESM1_ESM.docx]

| Table S1. Characteristics of patients who suffered from MACE | | | | | | | | | | |
| --- | --- | --- | --- | --- | --- | --- | --- | --- | --- | --- |
| Age(yrs) | Gender | Circumstances | One of the 4-high-risk clinical items | Cardiac history | Voltage  (Volts) | Initial loss of consciousness | Burns injury (degree [%TBSA]) | Other symptoms | Initial ECG | Troponin assay |
| 9 | Male | kite against power line | Yes | No | High ≥1000V | Yes | No | No | Anormal | Normal |
| 74 | Male | work accident | Yes | Yes | Low <1000V | Yes | No | Headache, limb trauma | Anormal | Elevated |
| 18 | Male | domestic accident | Yes | No | Low <1000V | Yes | Yes (3 [1%]) | No | Anormal | Elevated |
| 88 | Male | domestic accident | Yes | Yes | Low <1000V | No | No | Chest pain | Normal | Elevated |
| 68 | Male | domestic accident | Yes | Yes | Low <1000V | No | No | No | Anormal | Elevated |
| 60 | Female | domestic accident | Yes | No | Low <1000V | No | Yes (1 [1%]) | Limb trauma | Anormal | Elevated |
