# Supplementary material for: Use of troponin assay after electrical injuries: a 15-year multicentre retrospective cohort in emergency departments
Source: Scand J Trauma Resusc Emerg Med. 2021 Sep 26;29:141. doi: 10.1186/s13049-021-00955-6 (PMC8474711; doi:10.1186/s13049-021-00955-6)
Supplement: Supplementary file 2 — Additional file 2: Table S2. Management and outcomes of patients according to their risk classification*. [file 13049_2021_955_MOESM2_ESM.docx]

| Table S2. Management and outcomes of patients according to their risk classification* | | |
| --- | --- | --- |
|  | Low risk patients  N= 573 (%) | High risk patients  N = 181 (%) |
| *Management* |  |  |
| Troponin assay | 380 (66.3) | 153 (84.5) |
| Elevated troponin | 4 (0.7) | 13 (7.2) |
| Second troponin assay | 119 (20.8) | 78 (43.1) |
| Troponin rise | 1 (0.2) | 13 (7.2) |
| Hospitalisation | 268 (46.8) | 136 (75.1) |
| Discharge from the ED | 305 (53.2) | 45 (24.9) |
| *Outcomes* |  |  |
| Cardiac Event | 4 (0.7) | 9 (5.0) |
| MACE | 0 | 6 (3.3) |
| Death (all causes) | 0 | 3 (1.7) |
| *according to the 4 high-risk clinical items combined (with previous known heart disease and/or exposure to a high voltage of ≥ 1000 Volts and/or with initial loss of consciousness and/or an abnormal initial ECG), only patients with ECG at baseline were included. Data were missing for 31 patients (n=31/785, 3.9%) mainly concerning the initial loss of consciousness.  ED: Emergency Department; MACE: Major Adverse Cardiac Event | | |
